# Supplementary material for: Single-cell transcriptomic analysis reveals diversity within mammalian spinal motor neurons
Source: Nat Commun. 2023 Jan 3;14:46. doi: 10.1038/s41467-022-35574-x (PMC9810664; doi:10.1038/s41467-022-35574-x)
Supplement: Supplementary file 1 — Supplementary Information [file 41467_2022_35574_MOESM1_ESM.docx]

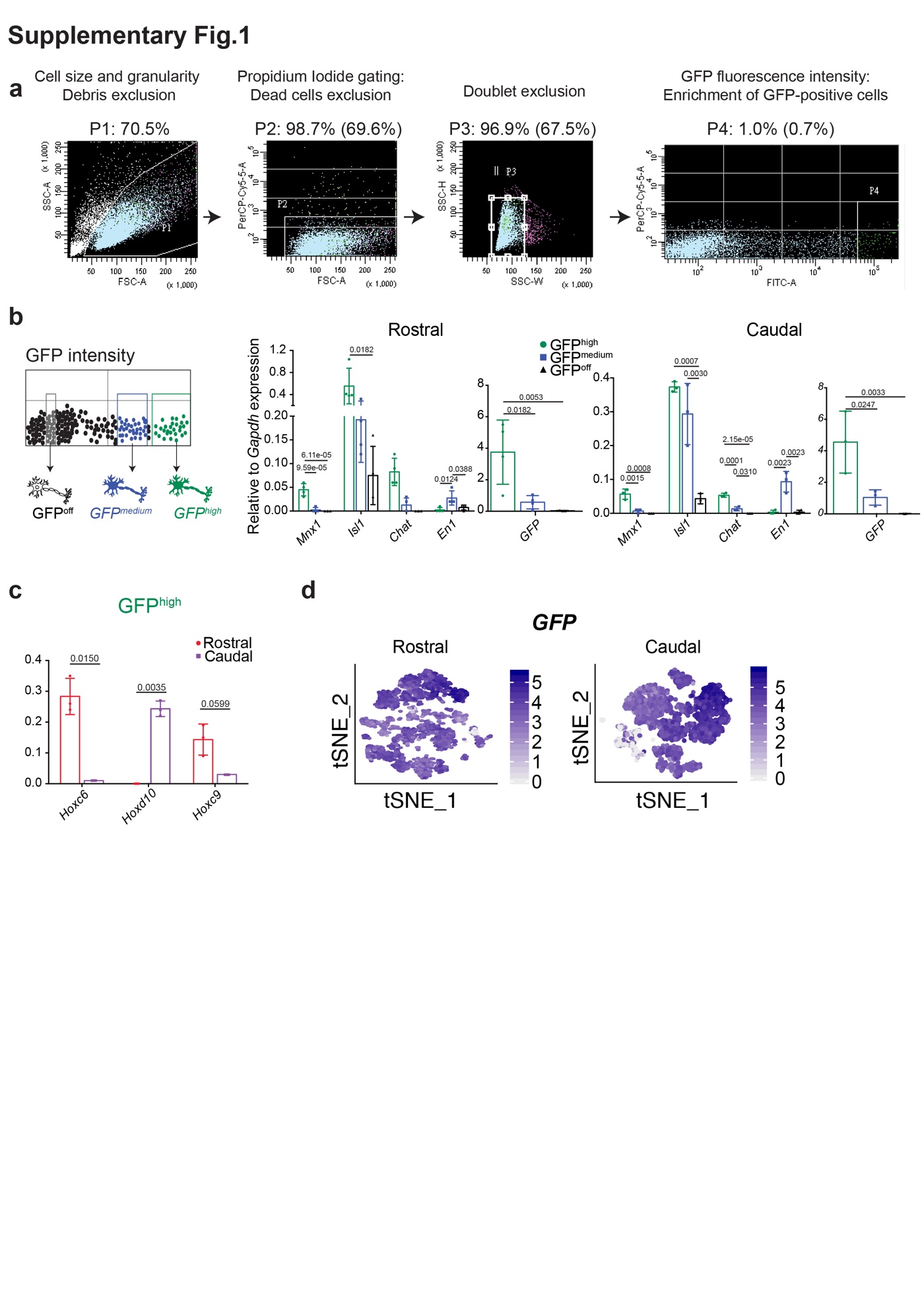


**Supplementary Fig. 1: FACS gating strategy and bulk qPCR analysis for dissociated spinal MNs.**

**a** Enrichment gating strategy based on the criteria: sample purity (P1), viability (P2), singlet or multiplets (P3), enrichment (P4) and yield. Percentages of desired populations are indicated above each plot, and total populations after subsequent gating are shown in parentheses. **b** Expression levels of MN markers and *GFP* genes relative to *Gapdh* from samples displaying differing GFP intensities (illustrated in the schematic diagram at left), as measured by RT-qPCR. **c** Expression levels of *Hox* genes to reflect segmental identities of the GFP^high^ sample. **d** tSNE map showing that *GFP* transcript was detected in most of the cells from rostral (left) and caudal (right) samples.

Mean ± SD, adjusted *p*-values are from **b**: One-way ANOVA with Tukey’s multiple comparison test, biological independent replicates *n*=4 for rostral, *n*=3 for caudal samples; **c**: Two-tailed paired t-test, *n*=3 biological independent replicates. Only significant values (adjusted *p*-value <0.05) are indicated. Source data are provided as a Source Data file.


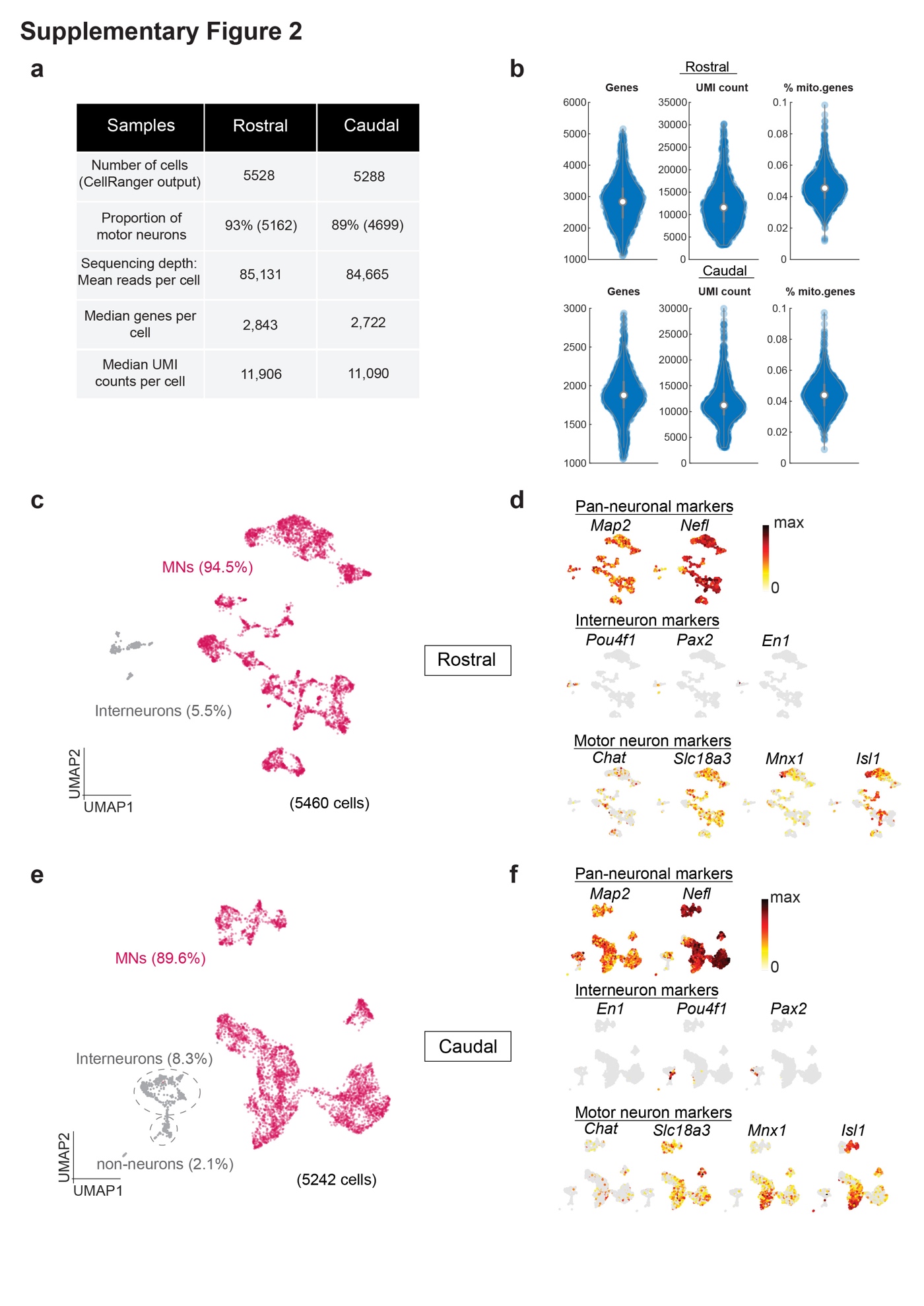


**Supplementary Fig. 2: Quality control of collected samples and isolated MNs from all sequenced cells.**

**a** Summary of median gene numbers, UMI counts, and cell numbers collected for each sample, based on CellRanger output. **b** Distribution of gene numbers and UMI counts, as well as the percentages of mitochondrial genes in the sequenced cells collected after removing low-quality cells. (Rostral: *n* = 5460 cells; Caudal: *n* = 5242 cells). The white open circles indicate median values, and the thin grey lines extend to the most extreme values within 1.5 times the IQR of the median. The width of filled color represents a density estimate of the distribution of values along the y-axis. **c** UMAP visualization of all cells from the rostral sample. Cells are grouped into MNs (red) or interneurons and non-neurons (grey) based on expression patterns of known markers shown in **d**. Percentages of each cell population in each sample are shown in parentheses. **d** UMAP distributions for expression in rostral samples of pan-neuronal, representative interneurons and MN markers. **e and f** as in **c and d**, except that caudal samples, were analysed. Single-cells were pooled from *n* = 12 embryos from 2 pregnant mice, rostral and caudal segments were separately processed.


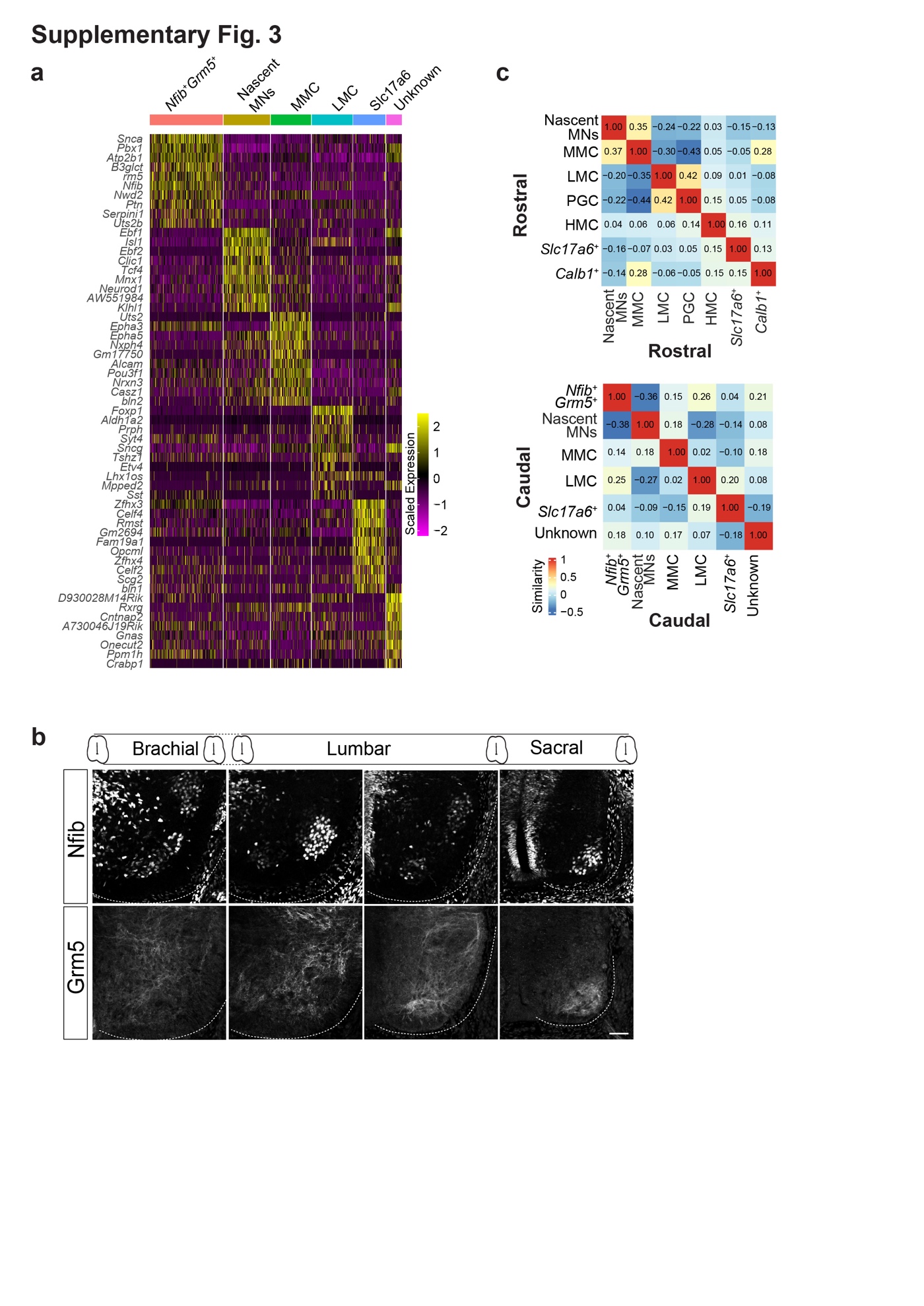


**Supplementary Fig. 3: Subtype annotation based on differentially expressing markers.**

**a** Heatmap showing the top 10 differentially expressing markers between major clusters in the caudal sample. **b** Protein expression of Nfib and Grm5 on adjacent slides showing that co-expressing cells are mainly present in the sacral segments. Immunostainings were repeated on *n*=5 embryos. Scale bar represents 50 μm. **c** Similarity analysis between clusters from rostral (top) and caudal (bottom) MNs.

**
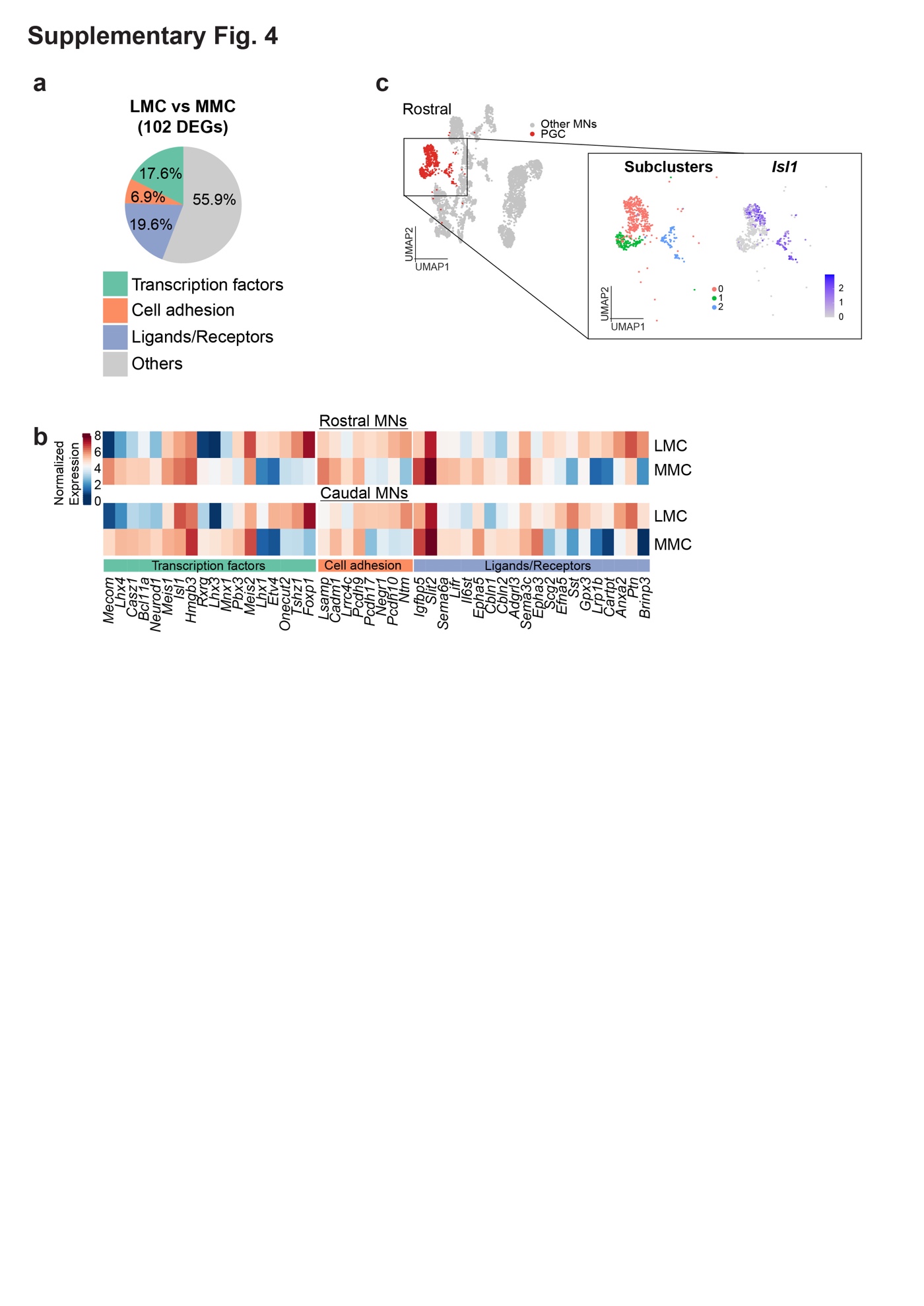
Supplementary Fig. 4: Differential gene expression between and within major MN subtypes.**

**a** Percentages of TFs, cell adhesion molecules, ligand/receptors and others in the DEGs identified from MMC and LMC MNs. **b** Heatmap reflecting normalized expression of the top-ranked DEGs between LMC and MMC neurons in different categories. **c** UMAP distribution of PGC MNs (red) in our single-cell dataset (left). Differential *Isl1* expression within the PGC clusters suggests a heterogeneity within PGC (right).

**
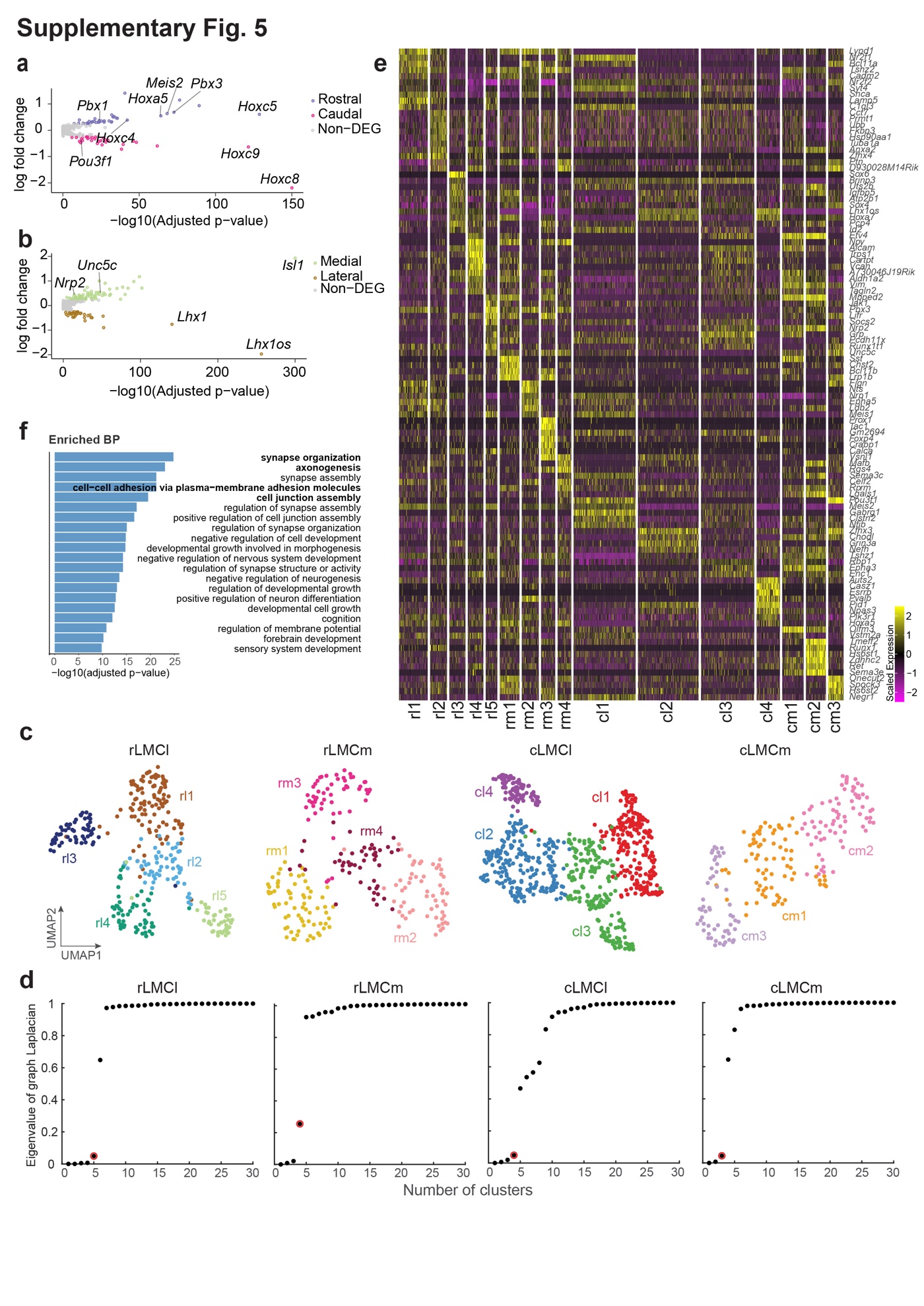
Supplementary Fig. 5: Sub-clustering analysis reveals heterogeneity among brachial LMC MNs.**

**a and b** Volcano plots showing the DEGs between **a** rostrocaudal and **b** mediolateral brachial LMC neurons. Genes with significance of adjusted *p*-value <0.05 and log fold-change >0.25 are color-labeled. Other non-significant genes are indicated by grey dots. Genes reported from other published studies have been labeled. **c** UMAP visualization of cellular heterogeneity within each “spatial quadrant” from brachial LMC MNs. Cells are colored based on clustering results as described in the Methods. **d** Eigenvalue spectra reveal the number of subclusters in each
“spatial quadrant”. The inferred number of clusters is marked in red, which is represented by the largest number before the largest eigenvalue gap. **e** Heatmap showing scaled expression of the top 10 markers for each subcluster. **f** Gene ontology enrichment of biological processes for DEGs across brachial LMC neurons. Terms of interest in this study are highlighted in bold. **a, b, f:** Adjusted *p*-values are from one-sided hypergeometric test followed by a Benjamini–Hochberg correction. r: rostral; c: caudal; m: medial; l: lateral.

**
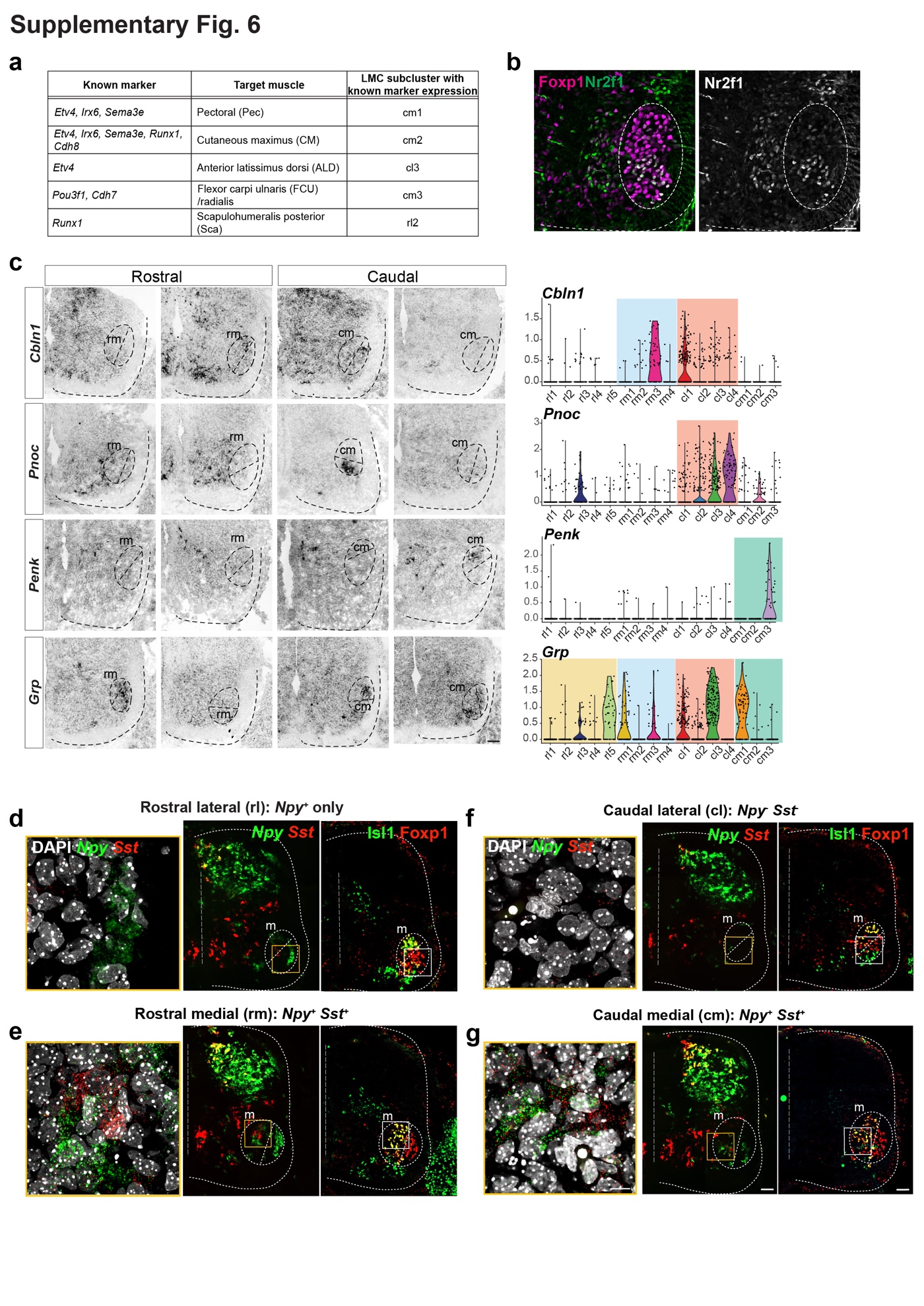
Supplementary Fig. 6: Differential expression of TFs and neuropeptides in brachial LMC MNs.**

**a** Table summarizes motor pool markers and their corresponding muscle targets from previous publications. LMC subclusters that express known motor pool markers are listed accordingly.  **b** Another example of differential TF expression, i.e., *Nr2f1* in a subset of LMC MNs, with reference to Fig. 4f. **c** *In situ* hybridization of selected neuropeptide genes (left) to validate our scRNA-seq results (right). **d-g** Detection of *Npy* and *Sst* expression in LMC MNs from each “spatial quadrant” using RNAscope technology. Rostral lateral shows *Npy*-only expression, rostral and caudal medial exhibit co-expression of *Npy* and *Sst*, whereas caudal lateral does not express either of these markers. Dashed contours outline the spinal cord boundary and spinal cord midline. Dashed circles define LMC MN positions, and the straight dashed white lines within the LMC demarcate the medial from lateral LMC MNs, based on immunostaining for spatial markers on adjacent sections. m = medial. Scale bar for the high-magnification image is 10 μm. Scale bar for image showing hemisection spinal cord represents 50 μm. Immunostaining and *in situ* hybridization of each marker were performed on *n*=3 embryos.

**
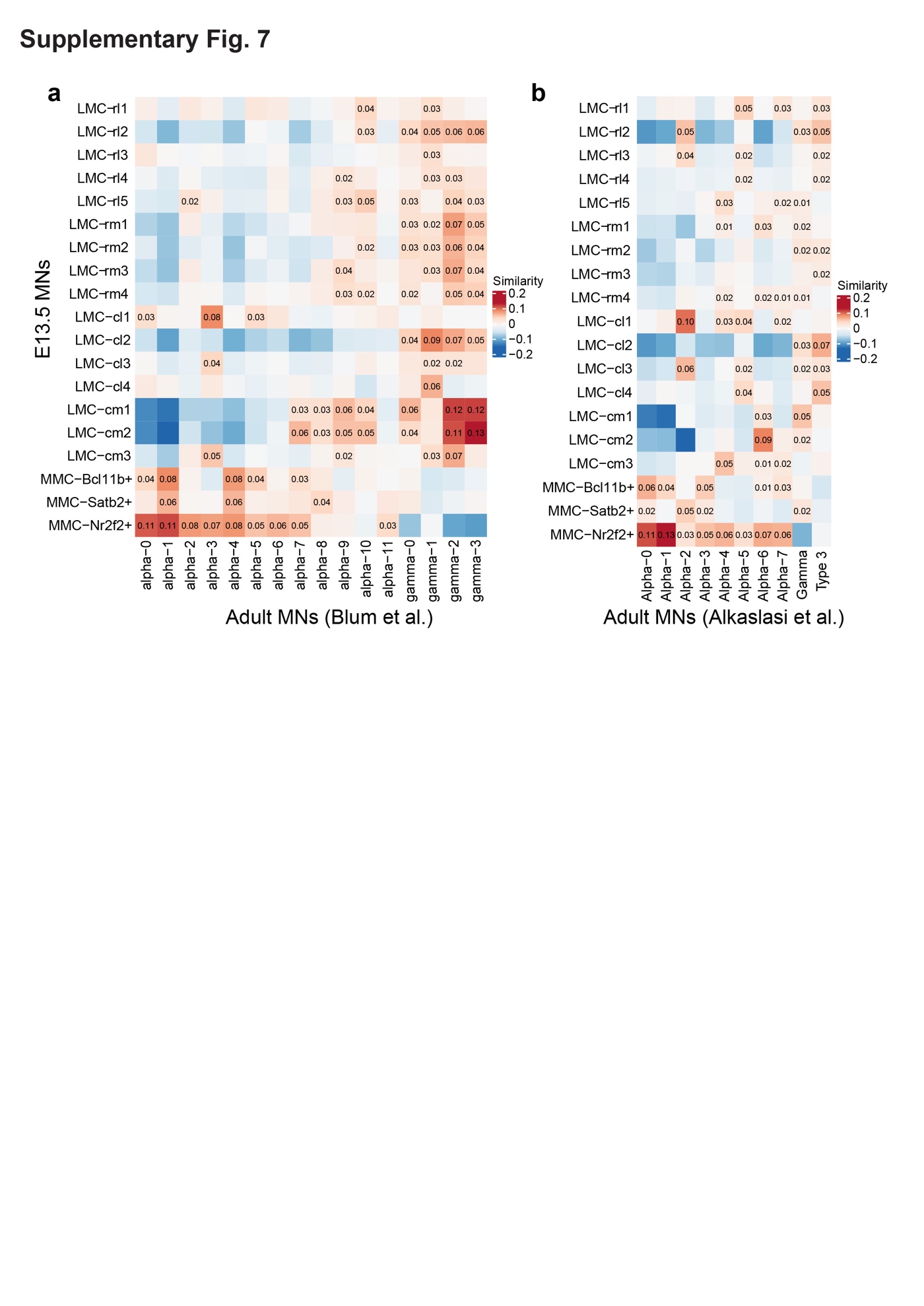
Supplementary Fig. 7: Similarity comparison of embryonic and adult MN subtypes.**

**a and b** Cluster comparison of LMC and MMC subclusters in our study with adult MN clusters from the **a** Blum *et al.*^13^ and **b** Alkaslasi *et al.*^14^ datasets, using CIDER for similarity analysis of differentially expressing genes.

**
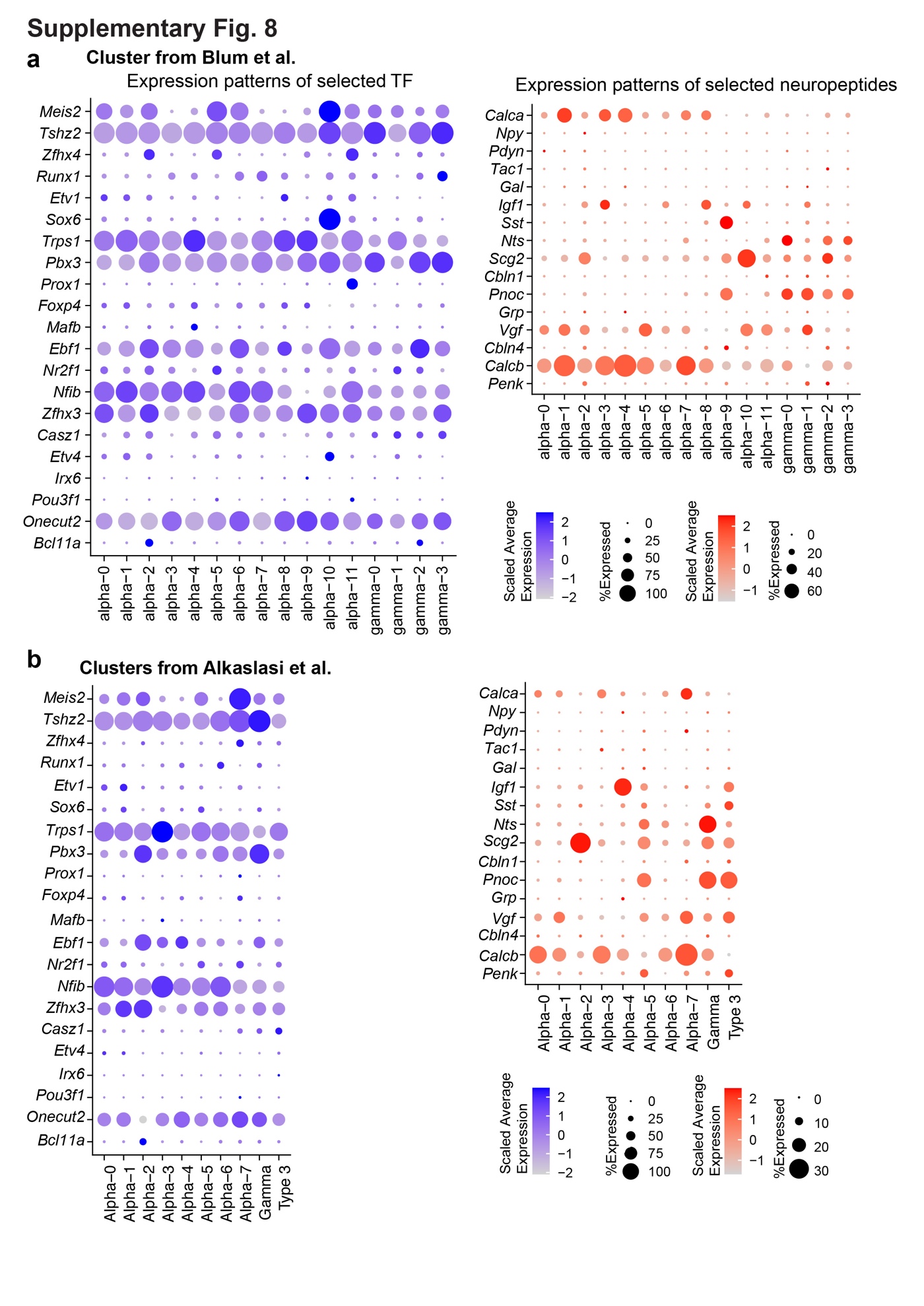
**

**Supplementary Fig. 8: Gene expression pattern of embryonic LMC subtype markers in adult spinal MNs.**

**a and b** Dot-plots showing the expression pattern of embryonic subtype-specific TFs and neuropeptides in adult MN clusters reported in the **a** Blum *et al*.^13^ and **b** Alkaslasi *et al*.^14^ datasets, respectively.

**
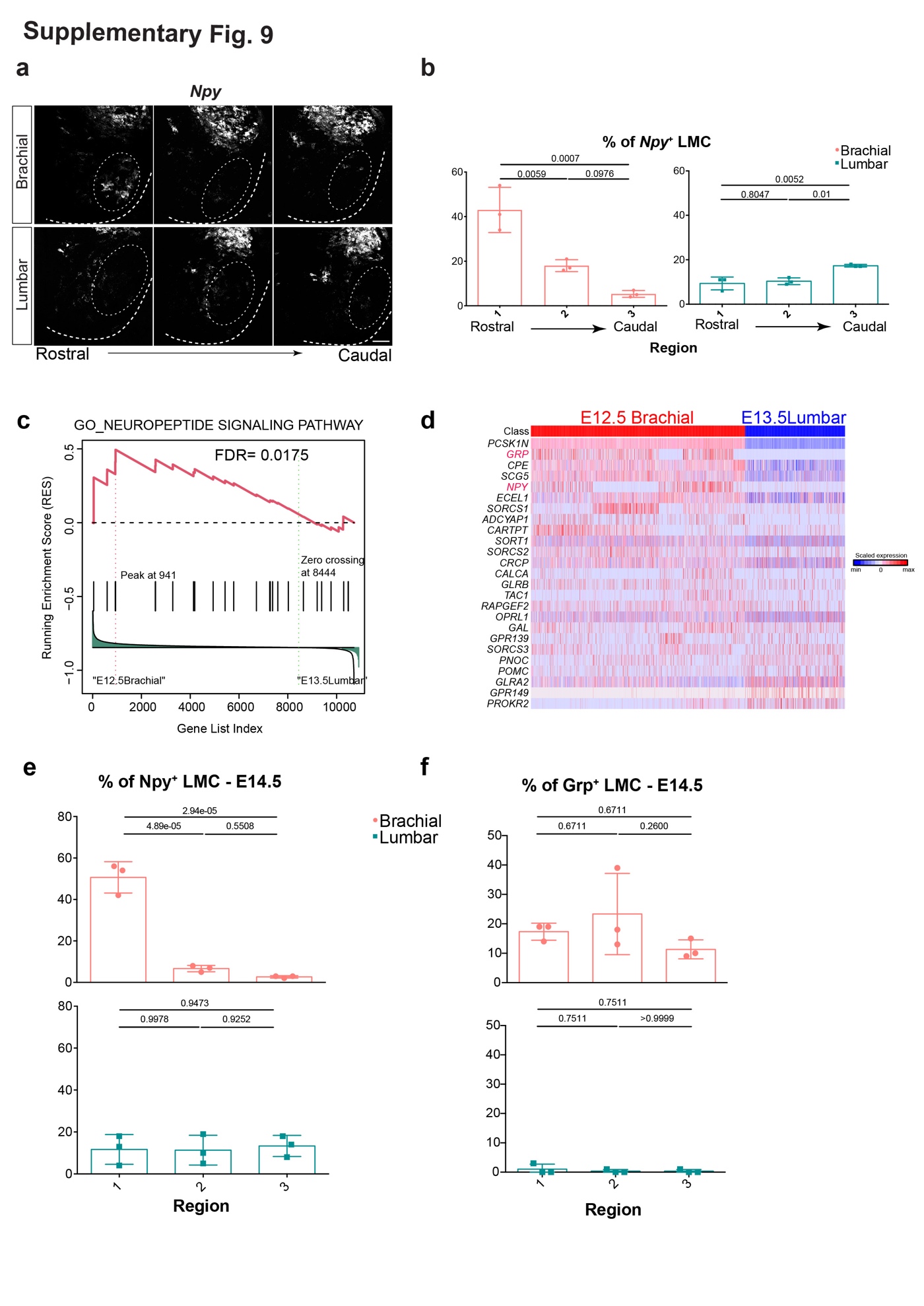
Supplementary Fig. 9: Comparison of LMC MNs from brachial and lumbar segments reveals differentially expressed neuropeptides genes.**

**a** Fluorescence ISH of the brachial-enriched *Npy* neuropeptides. Images are arranged from left to right, according to their relative position along the rostrocaudal axis of brachial (upper panel) and lumbar (bottom panel) spinal cord. Scale bar represents 50 μm. **b** Quantification of the percentage of *Npy*^+^ cells in LMC MNs according to their relative position from rostral to caudal (1🡪 3) within the brachial (salmon circle) and lumbar (turquoise square) segment. **c** GSEA plot showing enrichment of genes related to the neuropeptide signaling pathway in E12.5 brachial LMC MNs relative to E13.5 lumbar LMC MNs. False discovery rate (FDR) is the estimated probability that the normalized enrichment score represents a false positive finding. **d** Heatmap showing the differentially expressed genes from the gene list in **c**. **e and f** Quantification of the percentage of neuropeptide-expressing cells for **e** *Npy* and **f** *Grp* in Chat^+^ or Slc18a3^+^ LMC MNs of the E14.5 brachial and lumbar spinal cord. **b, e, f:** Adjusted *p* values were calculated by one-way ANOVA with Tukey’s multiple comparison test, *n* = 3 embryos. Data are presented as mean ± SD. Source data are provided as a Source Data file.

**
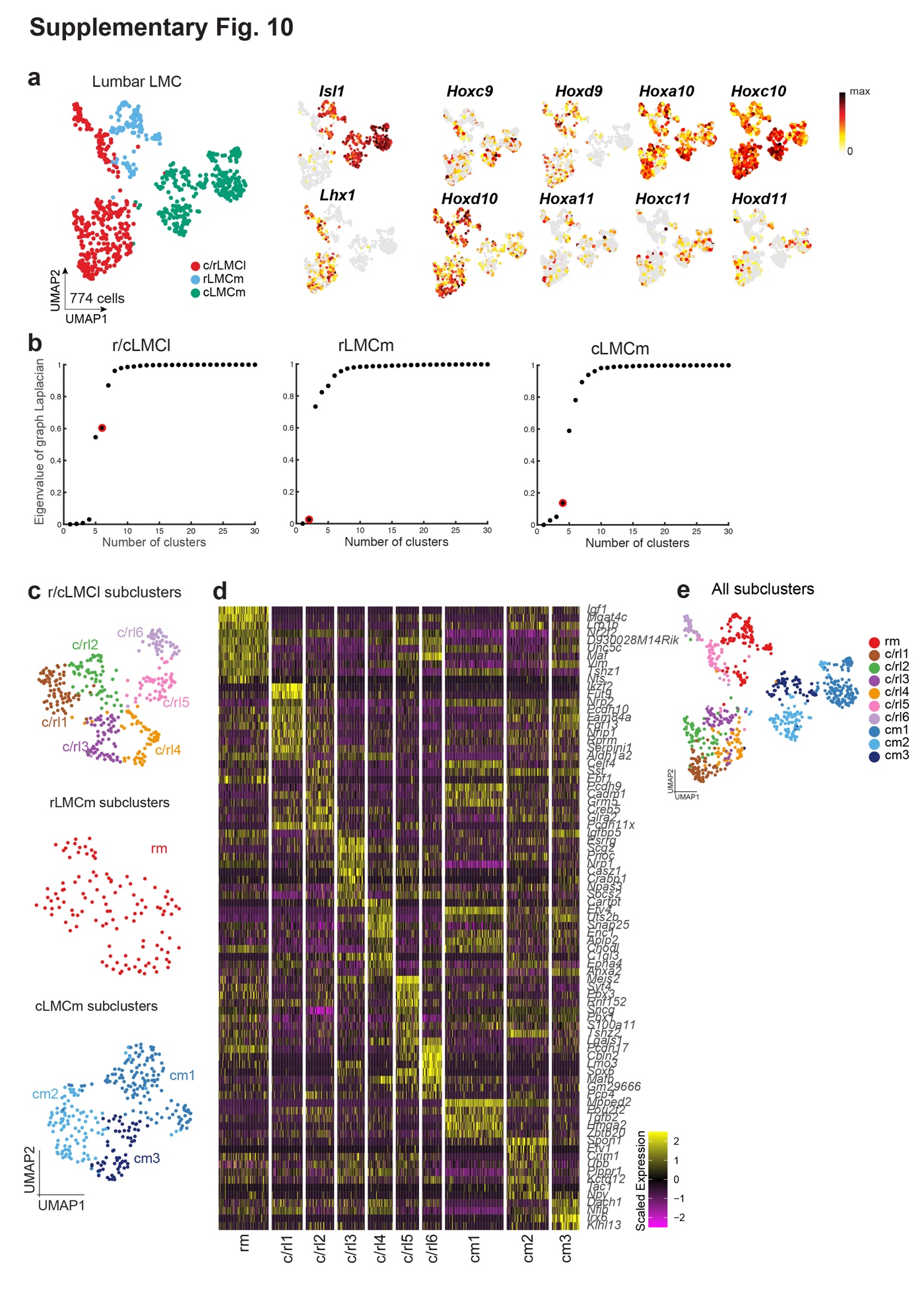
Supplementary Fig. 10: Sub-clustering analysis reveals heterogeneity among lumbar LMC MNs.**

**a** UMAP visualization of all lumbar LMC MNs. Cells have been grouped into
“spatial quadrants” based on the expression of *Hox* (rostral: Hoxd10^+^; caudal: Hoxd10^-^) and *LIM* homeodomain genes (medial: *Isl1*; lateral: *Lhx1*). Note, among lateral LMC MNs, cells could not be separated into caudal and rostral lumbar segments, so they have been annotated as “crl”. r: rostral; c: caudal; m: medial; l: lateral. **b** Eigenvalue spectra reveal the number of subclusters in each “spatial quadrant”. The inferred number of clusters is marked in red. **c** UMAP visualization of cellular heterogeneity within each “spatial quadrant” of lumbar LMC MNs. Cells have been colored according to identified subclusters. **d** Heatmap demonstrating scaled expression of the top 10 markers for each subcluster. **e** UMAP distribution of all LMC subclusters. Cells have been color-coded according to the clustering results in **c**. Single-cells were pooled from *n* = 12 embryos from 2 pregnant mice.


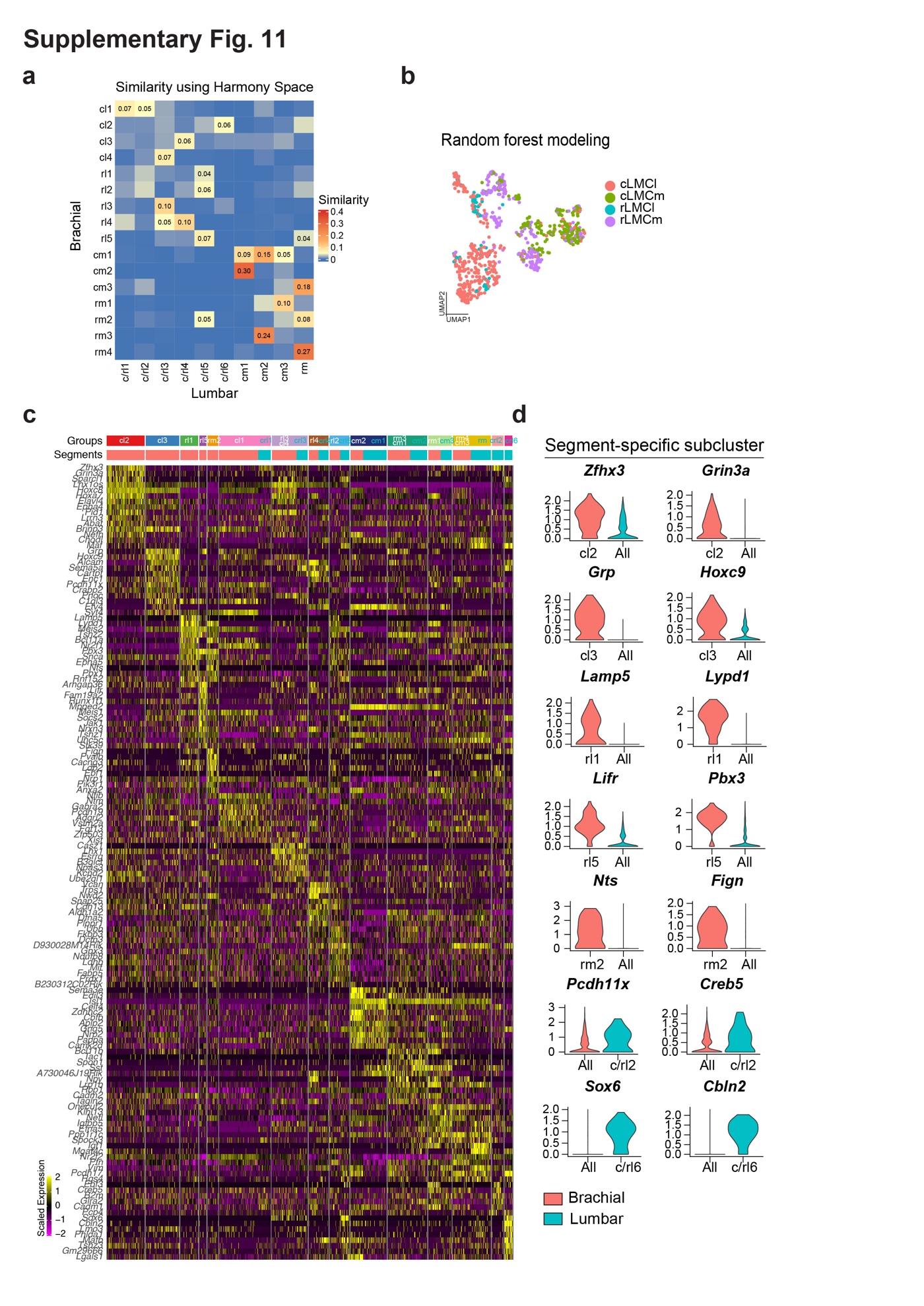


**Supplementary Fig. 11: Comparison of LMC subclusters between limbs identified both molecularly shared and specific subtypes.**

**a** Similarity analysis between LMC subclusters from rostral and caudal segments, as reflected by the heatmap, was quantified by the proportion of overlapping mutual nearest neighbors in the corrected PCA space using the Harmony algorithm. **b** Assignment of cell identities for lumbar LMC MNs based on highly variable genes and cell identities from brachial LMC MNs using a random forest modeling approach. In comparison to our unbiased clustering results shown in Supplementary Fig. 10a, this approach enabled segregation between lateral and medial LMC MNs, but failed to distinguish rostral and caudal cell identities. **c** Heatmap illustrating scaled expression of the top-ranked marker genes (rows) for cells in each group (columns) after merging brachial and lumbar LMC subclusters. Top (‘Groups’) bar labels subclusters from the merged dataset. The second ‘Segments’ bar identifies cells from brachial (salmon) and lumbar (turquoise) samples. **d** Violin plot showing top marker gene expression from segment-specific subclusters against all cells (All) from brachial or lumbar segments. Salmon: brachial; turquoise: lumbar.


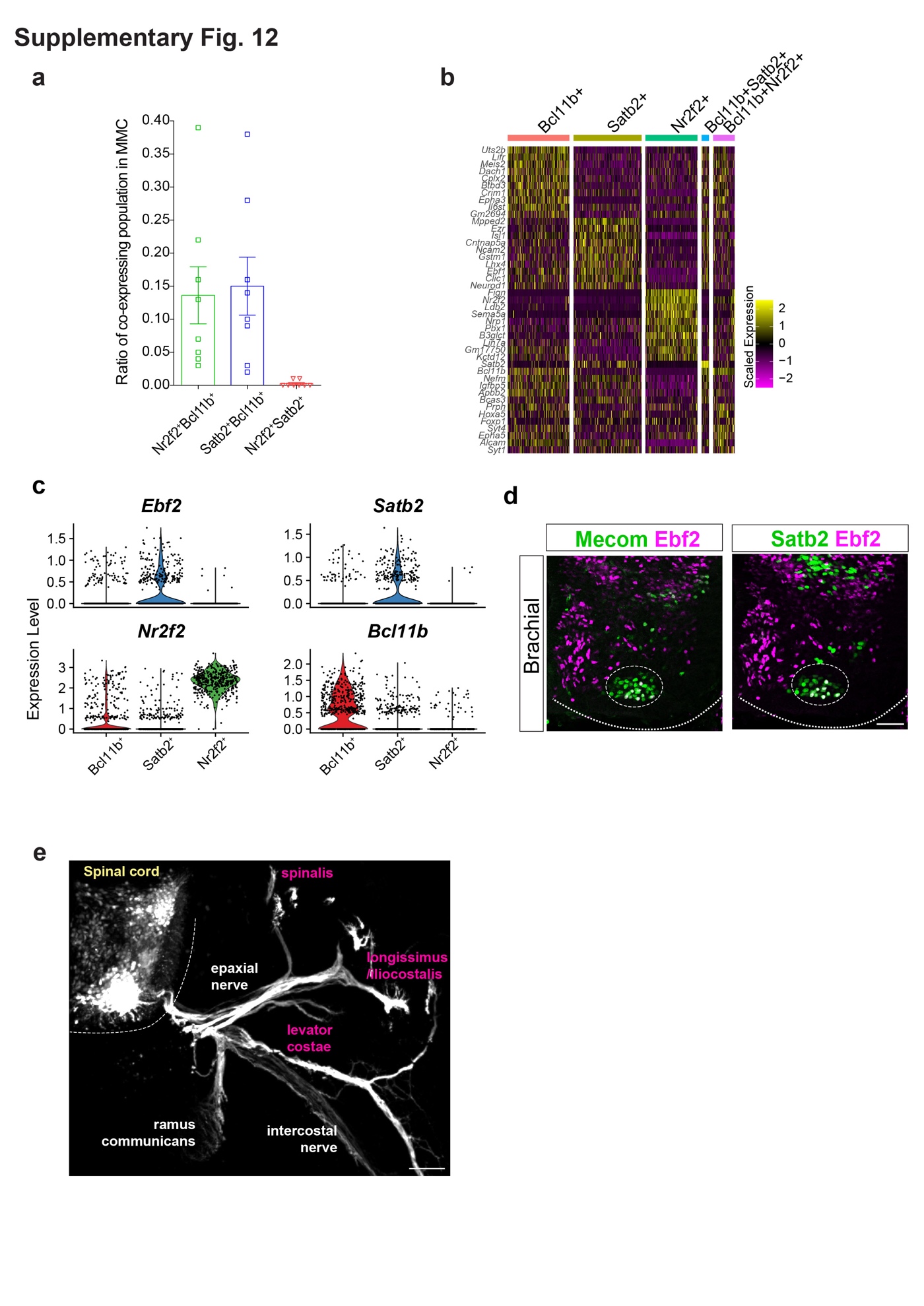


**Supplementary Fig. 12: Molecular heterogeneity among MMC neurons.**

**a** Quantification of the ratios of Bcl11b^+^:Satb2^+^-, Bcl11b^+^:Nr2f2^+^-, or Nr2f2^+^:Satb2^+^-co-expressing cells to total cells in the MMC region of the spinal cord. **b** Heatmap showing the top genes that are differentially expressed between MMC subtypes, and the co-expressing cells. Co-expressing cells share similar marker expression patterns with three major MMC subtypes. **c and d** **c** scRNA gene expression and **d** immunostainings of Ebf2 and Satb2 reveal co-expression of *Ebf2* in a fraction of the *Satb2^+^* cells. **e** Epaxial nerve branches in the thoracic segment. Epaxial muscles projected by each branch are labeled with reference to the motor nuclei map of Smith and Hollyday^12^. Scale bar represents 50 μm. **a:** Results are shown as mean ± SD, representing average counts from *n*=8 embryos with ≥2 sections of brachial spinal cord per embryo, one-way ANOVA with Tukey’s multiple comparison test. All immunostaining was repeated on *n*=5 embryos. Source data are provided as a Source Data file.

**
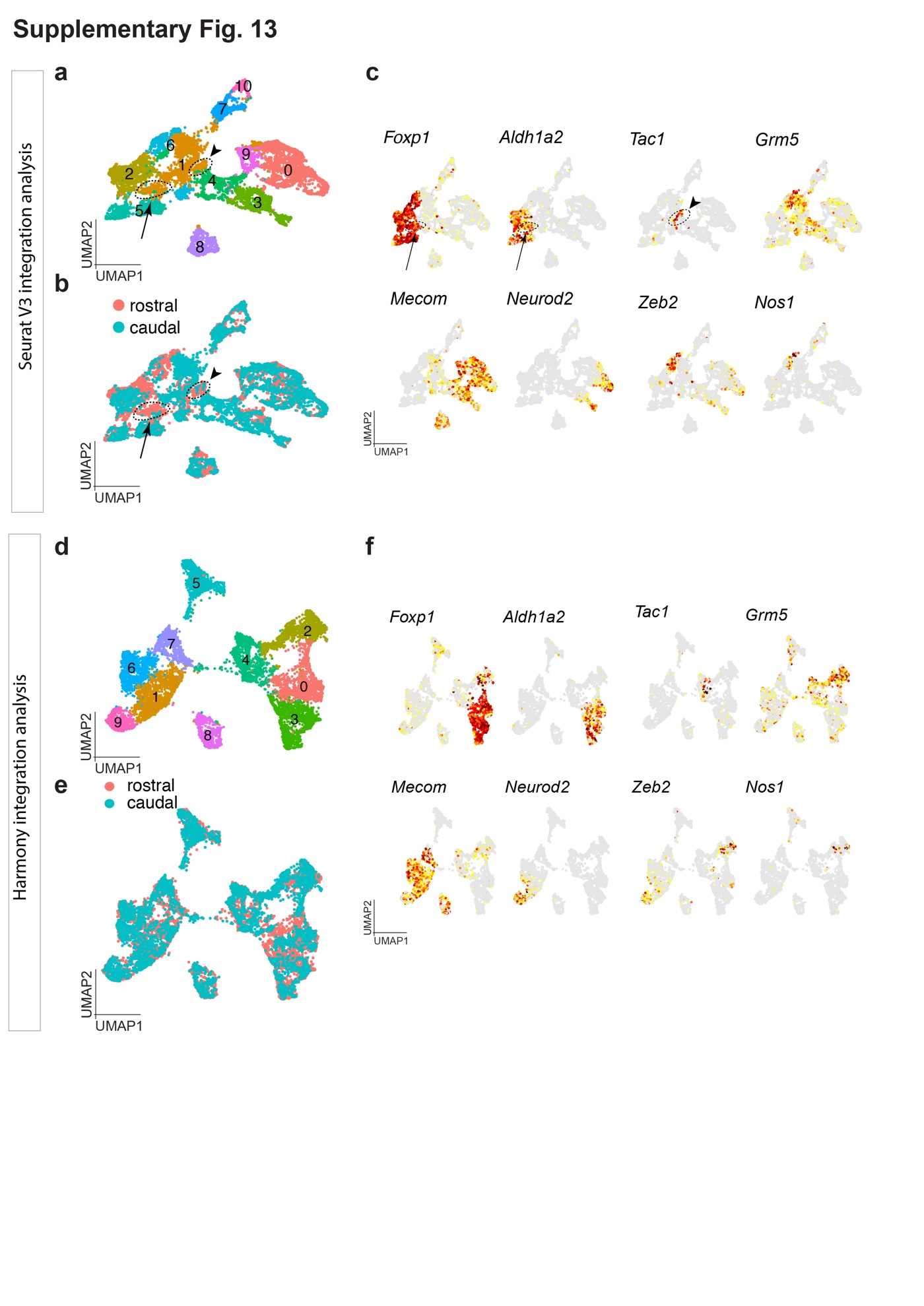
Supplementary Fig. 13: Integrative analysis of all cells from rostral and caudal samples using Seurat v3 (upper panel) and Harmony (bottom panel).**

**a and d** UMAP shows results from rough clustering upon integration. **b and e** UMAP shows cell distribution based on the origin of sample. **c and f** Marker gene expression distribution on UMAP (LMC: *Foxp1* and *Aldh1a2*; HMC: *Tac1*; Nfib^+^Grm5^+^: *Grm5*; MMC: *Mecom*; Nascent: *Neurod2*; PGC: *Zeb2* and *Nos1*). Arrow indicates incorrectly clustered LMC cells, arrowhead annotates the presumptive HMC cells. Single-cells were pooled from *n* = 12 embryos of 2 pregnant mice, rostral and caudal segments were separately processed.

Supplementary Table

| **Supplementary Table 1: In situ hybridization probe primer list used in this study (Related to Fig. 2e, 3c, 3d, 5, 6, 8g, 9c, Supplementary Fig. 6, 9).** | | |  |  |
| --- | --- | --- | --- | --- |
|  |  |  |  |  |
| **Gene** | **Forward primer** | **Reverse primer** | **Method of amplification** |  |
| Novel markers for PGC and HMC | | | |  |
|  |  |  |  |  |
| *Kitl* | AGCGTTTGGGATCATCTTTG | GAGTAATACGACTCACTATAGGGGGATCACTCCTAAGCCCACA | PCR |  |
| *Tac1* | GGTGGTAAGCTTGGTCTGACCGCAAAATCGAA | GGTGGTCTCGAGGTTCTGCATCGCGCTTCTTT | Cloning |  |
|  |  |  | F:HindIII-HF; R: XhoI |  |
| Differentially expressed MMC versus LMC genes | | | |  |
|  |  |  |  |  |
| *Ntm* | AACACCTCCACCAGCGTTAC | GAGTAATACGACTCACTATAGGGCAGTTGCTTGCTGTCCGTTA | PCR |  |
| *Lsamp* | ACTCAAAAGTGGCCTGGTTG | GAGTAATACGACTCACTATAGGGGGATCCGTTGATTCCTCTCA | PCR |  |
| Neuropeptides in LMC MNs | | | |  |
|  |  |  |  |  |
| *Pnoc* | ATGAAAATCCTCTTTTGT | GAGTAATACGACTCACTATAGGGTACACATTACCATTCTG | PCR |  |
| *Cbln1* | CGCTGGCTGTATTCCGTATT | GAGTAATACGACTCACTATAGGGACGAACCCCTTGAAAGTCCT | PCR |  |
| *Grp* | CCCGTAGGCAACAACATCCT | GAGTAATACGACTCACTATAGGGATCCCTTGCAGCTTCTTCCC | PCR |  |
| *Penk* | GAGACACCTTGGCCAACTCCT | GAGTAATACGACTCACTATATAGGGACAGCACAAAGCAGCATGTGA | PCR |  |
| *Igf1* | TGGATGCTCTTCAGTTCGTG | GAGTAATACGACTCACTATATAGGGCTGCACTTCCTCTACTTGTG | PCR |  |
| *Sst* | GGTGGTGGATCCACGCTACCGAAGCCGTC | GGTGGTCTCGAGGGGGCCAGGAGTTAAGGA | Cloning |  |
|  |  |  | F:BamHI-HF; R: XhoI |  |
| *Npy* | GGTGGTAAGCTTGGATCTCTTCTCTCACAGAGGC | GGTGGTCTCGAGCAACAACAACAAGGGAAATGG | Cloning |  |
|  |  |  | F: HindIII-HF; R:XhoI |  |
|  |  |  |  |  |
| **RNAScope** | | | |  |
| **Probe** | **Product Information** | **Target region** | **Number of pairs** |  |
| Mm-*Sst* | Cat# 404631 | 18 - 407 | 6 |  |
| Mm-*Npy*-C2 | Cat# 313321-C2 | 28 - 548 | 12 |  |
| Mm-*B3glct* | Cat# 440301 | 802 - 1757 | 20 |  |
| Mm-*Grm5*-C2 | Cat# 423631-C2 | 1199 - 2142 | 20 |  |
| Mm-*Nfib*-C3 | Cat# 586511-C3 | 1423 - 2372 | 20 |  |
| Mm-*Chat* | Cat# 408731 | 1090 - 1952 | 20 |  |
| Mm-*Slc18a3*-C3 | Cat# 448771-C3 | 1261 - 2405 | 20 |  |
| Mm-*Pou3f1*-C2 | Cat# 436421-C2 | 1365 - 2893 | 20 |  |
| Mm-*Penk*-C3 | Cat# 318761-C3 | 106 - 1332 | 20 |  |
| Mm-*Grp* | Cat# 317861 | 22 - 825 | 15 |  |
| Mm-*Etv4*-C2 | Cat# 458121-C2 | 256 - 1424 | 20 |  |
| Mm-*Igf1*-C3 | Cat# 443901-C3 | 359 - 1354 | 20 |  |
| Mm-*Nrp2*-C2 | Cat# 500661-C2 | 2001 - 2870 | 20 |  |
| Mm-*Satb2*-C3 | Cat# 413261-C3 | 647 - 1585 | 20 |  |
| Mm-*Nr2f2* | Cat# 480301 | 1532 - 3193 | 20 |  |
|  |  |  |  |  |
|  |  |  |  |  |
| Chick embryos | | | |  |
|  |  |  |  |  |
| *cNr2f2* | GGCATCGAGAACATCTGCGA | GAGTAATACGACTCACTATAGGGAAGATGCGGATGTGGTCCAT | PCR |  |
