# Supplementary material for: Single-cell transcriptomic analysis reveals diversity within mammalian spinal motor neurons
Source: Nat Commun. 2023 Jan 3;14:46. doi: 10.1038/s41467-022-35574-x (PMC9810664; doi:10.1038/s41467-022-35574-x)
Supplement: Supplementary file 3 — Description of Additional Supplementary Files [file 41467_2022_35574_MOESM3_ESM.pdf]

Description of Additional Supplementary Files

Title: Supplementary Data 1

Description: Marker genes for all a) rostral and b) caudal MN clusters (related to Fig. 1e and 1f). *P*-values are all from two-sided Wilcoxon rank sum tests followed by Bonferroni corrections.

Title: Supplementary Data 2

Description: Differentially expressed genes between a) rostral, and b) caudal LMC and MMC MNs (related to Supplementary Fig. 4a, 4b). *P*-values are all from two-sided Wilcoxon rank sum tests followed by Bonferroni corrections.

Title: Supplementary Data 3

Description: Marker genes for each a) brachial and b) lumbar LMC subcluster (related to Fig. 4, 5 Supplementary Fig. 5 and 10). *P*-values are all from two-sided Wilcoxon rank sum tests followed by Bonferroni corrections.

Title: Supplementary Data 4

Description: Differentially expressed genes between brachial and lumbar LMC MNs (related to Fig. 6).
