# Supplementary material for: Single-cell transcriptomic analysis reveals diversity within mammalian spinal motor neurons
Source: Nat Commun. 2023 Jan 3;14:46. doi: 10.1038/s41467-022-35574-x (PMC9810664; doi:10.1038/s41467-022-35574-x)
Supplement: Supplementary file 8 — Reporting Summary [file 41467_2022_35574_MOESM8_ESM.pdf]

nature portfolio

Corresponding author(s): Jun-An Chen, Qing Nie

Last updated by author(s): Nov 25, 2022

Reporting Summary

Nature Portfolio wishes to improve the reproducibility of the work that we publish. This form provides structure for consistency and transparency in reporting. For further information on Nature Portfolio policies, see our Editorial Policies and the Editorial Policy Checklist.

Statistics

For all statistical analyses, confirm that the following items are present in the figure legend, table legend, main text, or Methods section.

n/a	Confirmed	
☐	☒	The exact sample size (*n*) for each experimental group/condition, given as a discrete number and unit of measurement
☐	☒	A statement on whether measurements were taken from distinct samples or whether the same sample was measured repeatedly
☐	☒	The statistical test(s) used AND whether they are one- or two-sided<br>*Only common tests should be described solely by name; describe more complex techniques in the Methods section.*
☐	☒	A description of all covariates tested
☐	☒	A description of any assumptions or corrections, such as tests of normality and adjustment for multiple comparisons
☐	☒	A full description of the statistical parameters including central tendency (e.g. means) or other basic estimates (e.g. regression coefficient) AND variation (e.g. standard deviation) or associated estimates of uncertainty (e.g. confidence intervals)
☐	☒	For null hypothesis testing, the test statistic (e.g. *F*, *t*, *r*) with confidence intervals, effect sizes, degrees of freedom and *P* value noted<br>*Give P values as exact values whenever suitable.*
☒	☐	For Bayesian analysis, information on the choice of priors and Markov chain Monte Carlo settings
☒	☐	For hierarchical and complex designs, identification of the appropriate level for tests and full reporting of outcomes
☐	☒	Estimates of effect sizes (e.g. Cohen's *d*, Pearson's *r*), indicating how they were calculated

Our web collection on statistics for biologists contains articles on many of the points above.

Software and code

Policy information about availability of computer code

Data collection	FACS data were collected and analyzed using BDFACSAria v6.1.3.
Data analysis	Cell Ranger pipeline (version 2.1.1, 10x Genomics, RRID:SCR_017344);<br>Prism (version: 6.0, GraphPad, RRID:SCR_005375);<br>ZEN 2012 blue edition (Carl Zeiss, RRID:SCR_013672);<br>Imaris 9.5.1 (version 9.5.1, Bitplane, RRID:SCR_007370);<br>Image J (version 1.51m, NIH, RRID:SCR_003070);<br>Ilastik (version 1.3.3, Berg et al 2019, RRID:SCR_015246);<br>R and RStudio (The R Foundation; RRID: SCR_001905);<br>Adobe Illustrator 2020 (Adobe, RRID: SCR_010279);<br>Adobe Photoshop 2021 (Adobe, RRID:SCR_014199);<br>CIDER package (version 0.99.0, Hu et al 2021);<br>BD FACSDiva Software v6.1.3 (Becton Dickinson, RRID:SCR_001456).<br><br>R packages:<br>Seurat (version 2.3.4, version 3, Satija et al 2015, RRID:SCR_016341);<br>Harmony (version 1.0, Korsunsky et al 2019, https://github.com/immunogenomics/harmony);<br>UMAP (version 0.2.6.0, McInnes, Healy and Melville 2020, https://github.com/lmcinnes/umap);<br>clusterProfiler (version 3.14.3, Yu et al 2012, RRID: SCR_016884);

ComplexHeatmap (version 2.7.1., Gu et al 2016, https://github.com/jokergoo/ComplexHeatmap);
ggplot2 (version 3.3.2, https://cran.r-project.org/web/packages/ggplot2/index.html, RRID:SCR_014601)
ggalluvial (version 0.12.3, https://www.rdocumentation.org/packages/ggalluvial/versions/0.12.3)

The codes used for scRNA-seq data analysis were an adaptation of standard R packages, as described in the Methods section. The computational codes and metadata are available on GitHub (https://github.com/sqjin/scRNA-seq-motor-neuron) and Zenodo repositories (https://zenodo.org/record/7374602#.Y4WcUy2B060).

For manuscripts utilizing custom algorithms or software that are central to the research but not yet described in published literature, software must be made available to editors and reviewers. We strongly encourage code deposition in a community repository (e.g. GitHub). See the Nature Portfolio guidelines for submitting code & software for further information.

Data

Policy information about availability of data

All manuscripts must include a data availability statement. This statement should provide the following information, where applicable:

- Accession codes, unique identifiers, or web links for publicly available datasets
- A description of any restrictions on data availability
- For clinical datasets or third party data, please ensure that the statement adheres to our policy

The E13.5 mouse spinal MNs scRNA-seq raw data generated in this study have been deposited in the Gene Expression Omnibus repository under accession code GSE183759: https://www.ncbi.nlm.nih.gov/geo/query/acc.cgi?acc=GSE183759. The public datasets of adult spinal MNs used in this study are available at http://spinalcordatlas.org. All data supporting the findings of this study are available in within the paper and the Supplementary Information. Source data are provided with this paper.

Human research participants

Policy information about studies involving human research participants and Sex and Gender in Research.

Reporting on sex and gender	The sex of the human embryos were not determined at time of collection.
Population characteristics	Non-relevant in this study, as only one human embryo sample tissue is collected each for stage G.W.7.9 and 10.3.
Recruitment	Tissues were collected following abortion and women's written informed consent specifying the research that will be conducted, in accordance with legal procedures agreed by the national agency for biomedical research.
Ethics oversight	French Biomedicine Agency (authorization number PFS12-002 ; Agence de la Biomédecine).

Note that full information on the approval of the study protocol must also be provided in the manuscript.

Field-specific reporting

Please select the one below that is the best fit for your research. If you are not sure, read the appropriate sections before making your selection.

[x] Life sciences   [ ] Behavioural & social sciences   [ ] Ecological, evolutionary & environmental sciences

For a reference copy of the document with all sections, see nature.com/documents/nr-reporting-summary-flat.pdf

Life sciences study design

All studies must disclose on these points even when the disclosure is negative.

Sample size	No statistical methods were performed for sample size predetermination, sample size was determined iteratively. For scRNA-seq, 9861 cells were obtained from pooled n=12 embryos from 2 pregnant mice after quality control and allowed clustering into multiple distinct cell clusters and the detection of known MN subtypes. Sample size for each quantification analysis is detailed in the figure legends, where n = 3-10 embryos were used correspondingly in this study, which is comparable to previous study (Blum et al., 2021)
Data exclusions	For scRNA-seq data, cells were retained for subsequent analysis if they displayed a number of genes between 1000 and 5300, UMI counts <30500, as well as <10% mitochondrial counts. During initial clustering, non-motor neurons were filtered based on expression of known markers, retaining only our cells of interest, as detailed in the methods in the manuscript.
Replication	All attempts at replication were successful and can be performed independently on three biological replicates, except for validation on human spinal cord (n=1 each for GW7.9 and GW10.3 on 2-3 different sections) due to limited sample can be acquired and single-cell sample due to cost and logistics. To obtain single-cell MNs, E13.5 embryos of pregnant mice (n=12 embryos from two pregnant mice) were pooled to perform scRNA-seq. Rostral and caudal segments were separated by dissection and processed independently.

Randomization	scRNA-seq and validation experiment was performed on wild-type or reporter mice, thus randomization is irrelevant in this study.
Blinding	Single-cells were clustered unbiasedly based on transcriptome similarities and cluster identities were assigned based on expression of known markers. Investigator was not blinded because the quantification analysis and the assessment was performed in an objective and semi-automated manner.

Reporting for specific materials, systems and methods

We require information from authors about some types of materials, experimental systems and methods used in many studies. Here, indicate whether each material, system or method listed is relevant to your study. If you are not sure if a list item applies to your research, read the appropriate section before selecting a response.

Materials & experimental systems

n/a	Involved in the study	
☐	☒	Antibodies
☒	☐	Eukaryotic cell lines
☒	☐	Palaeontology and archaeology
☐	☒	Animals and other organisms
☒	☐	Clinical data
☒	☐	Dual use research of concern

Methods

n/a	Involved in the study	
☒	☐	ChIP-seq
☐	☒	Flow cytometry
☒	☐	MRI-based neuroimaging

Antibodies

Antibodies used	Primary antibodies: rabbit anti-Lhx3 (1:2000, Abcam Cat# ab14555, RRID:AB_301332); rabbit anti-Foxp1(1:20000, Abcam Cat# ab16645, RRID:AB_732428); goat anti-Foxp1(1:100 R&D systems Cat# AF4534, RRID:AB_2107102); rabbit anti-Hoxc8 (1:5000 Sigma-Aldrich Cat# HPA028911, RRID:AB_10602236); sheep anti-GFP (1:1000 AbD Serotec/Bio-Rad Cat# 4745–1051, RRID:AB_619712); rabbit anti-RFP (1:500 Abcam Cat# ab62341, RRID: AB_945213); mouse anti-MNR2/MNX1/HB9 (1:50 DSHB Cat# 81.5C10, RRI D:AB_2145209); goat anti-Chat (1:100 Millipore/Sigma Cat# ab144P, RRID:AB_2079751); mouse anti-COUP-TF2/NR2F2 (1:200 R&D systems Cat# PP-H7147-00, RRID:AB_2155627); mouse anti-COUP-TF1/NR2F1 (1:500 R&D systems Cat# PP-H8132-00, RRID:AB_2155494); rabbit anti-Evi1/Mecom (1:500 Cell Signaling Technology Cat# 2593, RRID:AB_2184098); goat anti-Isl1 (1:1000 Neuromics Cat# GT15051, RRID:AB_2126323); rabbit anti-Satb2 (1:1000 Abcam Cat# ab92446, RRID:AB_10563678); guinea pig anti-Satb2 (1:1000 Synaptic Systems Cat# 327004, RRID:AB_2620070); rat anti-Bcl11b/Ctip2 (1:1000 Abcam Cat# ab18465, RRID:AB_2064130); rabbit anti-Zfhx4 (1:200 Novus Cat# NBP1-82156, RRID:AB_11020060); rabbit anti-Calbindin D-28K/Calb1 (1:1000 Millipore/Sigma Cat# AB1778, RRID:AB_2068336); rabbit anti-Nfib (1:1000 Novus Cat# NBP1-81000, RRID:AB_11027763); rabbit anti-Grm5 (1:500 Millipore/Sigma Cat# AB5675, RRID:AB_2295173); sheep anti-Ebf2 (1:500 Novus Cat# AF7006, RRID:AB_10972102); rabbit anti-Nrp2 (1:200 Cell Signaling Technology Cat# 3366, RRID:AB_2155250). Guinea pig anti-Foxp1 (1:320000), anti-Isl1 (1:10000, cat# CU1277, RRID:AB_2631974), anti-Lhx1 (1:20000, cat# CU453, RRID:AB_2827967), anti-Unc5c (1:50), rabbit anti-Nkx6.1 (1:1000) were antibody gifts from Thomas Jessell and guinea pig anti-Mnx1 (1:1000) was gift from Hynek Wichterle. Guinea pig anti-Hoxa5 (1:20000, RRID:AB_2744661) was made in house.<br><br>Secondary antibodies were used at dilution titer of 1:1000: Donkey anti-Sheep IgG (H+L) Cross-Adsorbed, Alexa Fluor 488 (Thermo Fisher Scientific Cat# A-11015, RRID: AB_2534082); Donkey Anti-Goat IgG (H+L) Antibody, Alexa Fluor 488 (Thermo Fisher Scientific Cat# A-11055, RRID: AB_2534102); 488-AffiniPure Donkey Anti-Rat IgG (H+L) (Jackson ImmunoResearch Lab, Cat# 712005153, RRID: AB_2340631); Cy3-AffiniPure Donkey Anti-Guinea Pig IgG (H+L) (Jackson ImmunoResearch Lab, Cat# 706165148, RRID: AB_2340460); Cy3-AffiniPure Donkey Anti-Mouse IgG (H+L) (Jackson ImmunoResearch Lab, Cat# 706165150, RRID: AB_2340813); Cy3-AffiniPure Donkey Anti-Rabbit IgG (H+L) (Jackson ImmunoResearch Lab, Cat# 711165152, RRID: AB_2307443); Cy5-AffiniPure Donkey Anti-Guinea Pig IgG (H+L) (Jackson ImmunoResearch Lab, Cat# 706175148, RRID: AB_2340462); Cy5-AffiniPure Donkey Anti-Mouse IgG (H+L) (Jackson ImmunoResearch Lab, Cat# 715175150, RRID: AB_2340819); Cy5-AffiniPure Donkey Anti-Rabbit IgG (H+L) (Jackson ImmunoResearch Lab, Cat# 711175152, RRID: AB_2340607)
Validation	All commercially acquired antibodies used in this study were thoroughly validated by manufacturer and published studies. Individual antibody profiles and relevant citations were provided in the links.<br><br>rabbit anti-Foxp1: https://www.abcam.com/foxp1-antibody-ab16645.html<br>goat anti-Foxp1: https://www.rndsystems.com/products/human-foxp1-antibody_af4534<br>rabbit anti-Hoxc8: https://www.sigmaaldrich.com/TW/en/product/sigma/hpa028911<br>sheep anti-GFP: https://commerce.bio-rad.com/fetchCookies?mode=nonajax&returnURL=https://www.bio-rad-antibodies.com/polyclonal/green-fluorescent-protein-antibody-4745-1051.html?f=purified<br>rabbit anti-RFP: https://www.abcam.com/rfp-antibody-ab62341.html<br>mouse anti-MNR2/MNX1/HB9: https://dshb.biology.uiowa.edu/81-5C10<br>goat anti-Chat: https://www.merckmillipore.com/TW/zh/product/Anti-Choline-Acetyltransferase-Antibody,MM_NF-AB144P<br>mouse anti-COUP-TF2/NR2F2: https://www.rndsystems.com/products/human-coup-tf-ii-nr2f2-antibody-h7147_pp-h7147-00<br>mouse anti-COUP-TF1/NR2F1: https://www.rndsystems.com/products/human-coup-tf-i-nr2f1-antibody-clone-h8132-h8132_pp-h8132-00<br>rabbit anti-Evi1/Mecom: https://www.cellsignal.com/products/primary-antibodies/evi-1-c50e12-rabbit-mab/2593<br>goat anti-Isl1: https://www.neuromics.com/gt15051

rabbit anti-Satb2: https://www.abcam.com/satb2-antibody-epncir130a-ab92446.html
guinea pig anti-Satb2: https://sysy.com/product/327004
rat anti-Bcl11b/Ctip2: https://www.abcam.com/ctip2-antibody-25b6-ab18465.html
rabbit anti-Zfhx4: https://www.novusbio.com/products/zfhx4-antibody_nbp1-82156
rabbit anti-Calbindin D-28K/Calb1: https://www.sigmaaldrich.com/TW/en/product/mm/ab1778?cm_sp=Insite-_-caContent_prodMerch_cooccuranceModel-_-prodMerch10-3
rabbit anti-Nfib: https://www.novusbio.com/products/nfib-antibody_nbp1-81000
rabbit anti-Grm5: https://www.sigmaaldrich.com/TW/en/product/mm/ab5675
sheep anti-Ebf2: https://www.novusbio.com/products/ebf-2-antibody_af7006
rabbit anti-Lhx3: https://www.citeab.com/antibodies/742144-ab14555-anti-lhx3-lim-antibody
rabbit anti-Nrp2: https://www.cellsignal.com/products/primary-antibodies/neuropilin-2-d39a5-xp-rabbit-mab/3366

Donkey anti-Sheep IgG (H+L) Cross-Adsorbed, Alexa Fluor 488: https://www.thermofisher.com/antibody/product/Donkey-anti-Sheep-IgG-H-L-Cross-Adsorbed-Secondary-Antibody-Polyclonal/A-11015
Donkey Anti-Goat IgG (H+L) Antibody, Alexa Fluor 488: https://www.thermofisher.com/antibody/product/Donkey-anti-Goat-IgG-H-L-Cross-Adsorbed-Secondary-Antibody-Polyclonal/A-11055
488-AffiniPure Donkey Anti-Rat IgG (H+L): https://www.jacksonimmuno.com/catalog/products/712-005-153
Cy3-AffiniPure Donkey Anti-Guinea Pig IgG (H+L): https://www.jacksonimmuno.com/catalog/products/706-165-148
Cy3-AffiniPure Donkey Anti-Mouse IgG (H+L): https://www.jacksonimmuno.com/catalog/products/715-165-150
Cy3-AffiniPure Donkey Anti-Rabbit IgG (H+L): https://www.jacksonimmuno.com/catalog/products/711-165-152
Cy5-AffiniPure Donkey Anti-Guinea Pig IgG (H+L): https://www.jacksonimmuno.com/catalog/products/706-175-148
Cy5-AffiniPure Donkey Anti-Mouse IgG (H+L): https://www.jacksonimmuno.com/catalog/products/715-175-150
Cy5-AffiniPure Donkey Anti-Rabbit IgG (H+L): Cy5-AffiniPure Donkey Anti-Rabbit IgG (H+L) (Jackson ImmunoResearch Lab, Cat# 711175152

Information about antibody gifts
guinea pig anti-Isl1: https://scicrunch.org/resolver/AB_2631974
guinea pig anti-Lhx1: https://scicrunch.org/resolver/RRID:AB_2827967

Antibody made in-house
Hoxa5 peptide sequence: DSASMHSGRYGYGYN*C (* denotes cysteine used for KLH coupling), antibody generated based on Dasen et al., Cell, 2015 DOI:https://doi.org/10.1016/j.cell.2005.09.009). Western blot detects 3 bands between 30-50kDa.

Animals and other research organisms

Policy information about studies involving animals; ARRIVE guidelines recommended for reporting animal research, and Sex and Gender in Research

Laboratory animals	Mus musculus Mnx1-GFP, Npy-IRES Cre/Cre (JAX, 027851) males P50-180<br>Tau-mGFP (JAX, 021162), Mnx1-RFP, C57BL/6J females P50-150<br>All live animals were maintained in a C57BL/6J background and housed in the specific-pathogen-free (SPF) animal facility of Institute of Molecular Biology, Academia Sinica, with 12-h light/dark cycle, 45-55% humidity, 19-22oC temperature and had ad libitum access to food and water in their home cages at all times, abiding by the IACUC Academia Sinica guidelines.
Wild animals	No wild animals were used in this study.
Reporting on sex	Sex-based analysis was not performed because the profiling process is time-sensitive for genotyping on the embryonic samples.
Field-collected samples	No field-collected samples were used in this study.
Ethics oversight	All mice experimental procedures were performed in accordance to guidelines approved by the Institutional Animal Care and Use Committee (IACUC) at Academia Sinica (protocol number 19-12-1409 and 12-07-389). Human embryo spinal cords were harvested from material obtained following legally induced terminations of pregnancy (first trimester of pregnancy) from the Department of Obstetrics and Gynecology at the Antoine Béclère Hospital (Clamart, France). Fetal age was calculated by measuring the length of limbs and feet according to a developed mathematical model and none of the induced abortions were performed for reasons of fetal abnormality. Tissues were collected in absence of compensation, with written informed consent specifying the purpose of the research, in accordance with legal procedures agreed by the national agency for biomedical research (French Biomedicine Agency (authorization number PFS12-002; Agence de la Biomédecine).

Note that full information on the approval of the study protocol must also be provided in the manuscript.

Flow Cytometry

Plots

Confirm that:

- [x] The axis labels state the marker and fluorochrome used (e.g. CD4-FITC).
- [x] The axis scales are clearly visible. Include numbers along axes only for bottom left plot of group (a 'group' is an analysis of identical markers).
- [x] All plots are contour plots with outliers or pseudocolor plots.
- [x] A numerical value for number of cells or percentage (with statistics) is provided.

Methodology

Sample preparation	E13.5 mouse embryonic spinal cord was dissected at specific segments, enzymatically (papain) and mechanically (gentleMACS dissociator) dissociated into single cell suspension according to the manufacturer's protocol.
Instrument	BDFACSAria III cell sorter (BD BioSciences, USA)
Software	BD FACSDiva Software v6.1.3 (Becton Dickinson)
Cell population abundance	Post-sorted cell population with highest GFP intensity (motor neurons) have purity > =90%.
Gating strategy	Gating strategies were described in supplemental information (Supplementary Fig.1).

- [x] Tick this box to confirm that a figure exemplifying the gating strategy is provided in the Supplementary Information.
